# Incident Comorbidity, Resource Use, and All-Cause Mortality Associated with Prurigo Nodularis: A United Kingdom Retrospective Database Analysis

**DOI:** 10.1016/j.xjidi.2023.100233

**Published:** 2023-09-12

**Authors:** Christopher Ll Morgan, Melissa Thomas, Benjamin R. Heywood, Sonja Ständer, Shawn G. Kwatra, Zarif K. Jabbar-Lopez, Christophe Piketty, Sylvie Gabriel, Jorge Puelles

**Affiliations:** 1 Pharmatelligence, Cardiff, United Kingdom; 2 Department of Dermatology, University Hospital Münster, Münster, Germany; 3 Johns Hopkins Itch Center, Johns Hopkins University School of Medicine, Baltimore, Maryland, USA; 4 Galderma SA, Zug, Switzerland

## Abstract

We described comorbidity, resource utilization, and mortality for patients with prurigo nodularis (PN) using data from the Clinical Practice Research Datalink. Patients with incident PN (2008–2018) were selected and matched to controls. Of 2,416 patients with PN, 2,409 (99.7%) were matched to controls. Prevalence of atopic dermatitis (relative risk [RR] = 2.571; 95% confidence interval [CI] = 2.356–2.806), depression (RR = 1.705; 95% CI = 1.566–1.856), anxiety (RR = 1.540; 95% CI = 1.407–1.686), coronary heart disease (RR = 1.575; 95% CI = 1.388–1.787), chronic kidney disease (RR = 1.529; 95% CI = 1.329–1.759), and type 2 diabetes mellitus (RR = 1.836; 95% CI = 1.597–2.111) was significantly higher for patients with PN. Subsequent risk of atopic dermatitis (hazard ratio = 6.58; 95% CI = 5.17– 8.37), depression (hazard ratio = 1.61; 95% CI = 1.30–1.99), and coronary heart disease (hazard ratio = 1.37; 95% CI = 1.09–1.74) were significantly increased. Resource utilization was increased in all settings: incidence rate ratio = 1.48 (95% CI = 1.47–1.49) for primary care, incident rate ratio = 1.80 (95% CI = 1.75–1.85) for inpatients, incident rate ratio = 2.15 (95% CI = 2.13–2.18) for outpatients, and incidence rate ratio = 1.32 (95% CI = 1.27–1.36) for accident and emergency. Respective cost ratios were 1.78 (95% CI = 1.67–1.90), 1.52 (95% CI = 1.20–1.94), 2.34 (95% CI = 2.13–2.58), and 1.55 (95% CI = 1.33–1.80). Total primary and secondary healthcare costs were £2,531 versus £1,333, a cost ratio of 1.62 (95% CI = 1.36–1.94). The adjusted hazard ratio for mortality was 1.37 (95% CI = 1.14–1.66). Patients with PN had significantly increased rates of comorbidity, healthcare resources utilization, and mortality compared with matched controls.

## Introduction

Prurigo nodularis (PN) is a potentially debilitating inflammatory skin disease that has a major impact on patient QOL (Huang et al., 2020d; Tsianakas et al., 2016; Whang et al., 2022). Despite this, there is a lack of evidence concerning the resource use associated with the condition in England. In a previous study (Morgan et al., 2022), the prevalence of PN was estimated as 3.27 per 10,000 population, equivalent to 18,471 patients living with the condition in England. These patients also had relatively high occurrences of certain a priori selected comorbidities. This confirmed research from other settings that report associations between PN and a wide range of other comorbidities, including HIV, non-Hodgkin lymphoma, inflammatory skin conditions, metabolic syndrome manifestations, chronic kidney disease (CKD), heart failure, and various mental health disorders (Aggarwal et al., 2021; Huang et al., 2020b; Ständer et al., 2020).

Owing to the severe impact of the condition, there is considerable secondary care involvement in the management of patients with PN. A study across 14 countries, including the United Kingdom, found that although clinicians reported seeing relatively few patients with PN, reflecting the comparative rareness of the condition, 68% of patients with PN visited a clinician between two and four times per year (Pereira et al., 2018). A study in the United States (US) reported that over a third of patients with PN visited a physician at least 10 times in the previous year (Aggarwal et al., 2021), whereas a US cross-sectional study reported that PN accounted for 3.7 inpatient visits per 100,000 discharges and that patients with PN had a longer length of stay and higher costs associated with inpatient care (Whang et al., 2019).

In this study, we wished to explore further the apparently high prevalence of conditions that we previously reported by comparing rates with those of age- and gender-matched controls both at the time of diagnosis and the subsequent incidence of these conditions in the period after diagnosis. We also wish to compare overall primary and secondary care resource use and associated costs as well as patterns of mortality.

## Results

### Selected patients and baseline characteristics

**Figure 1:**
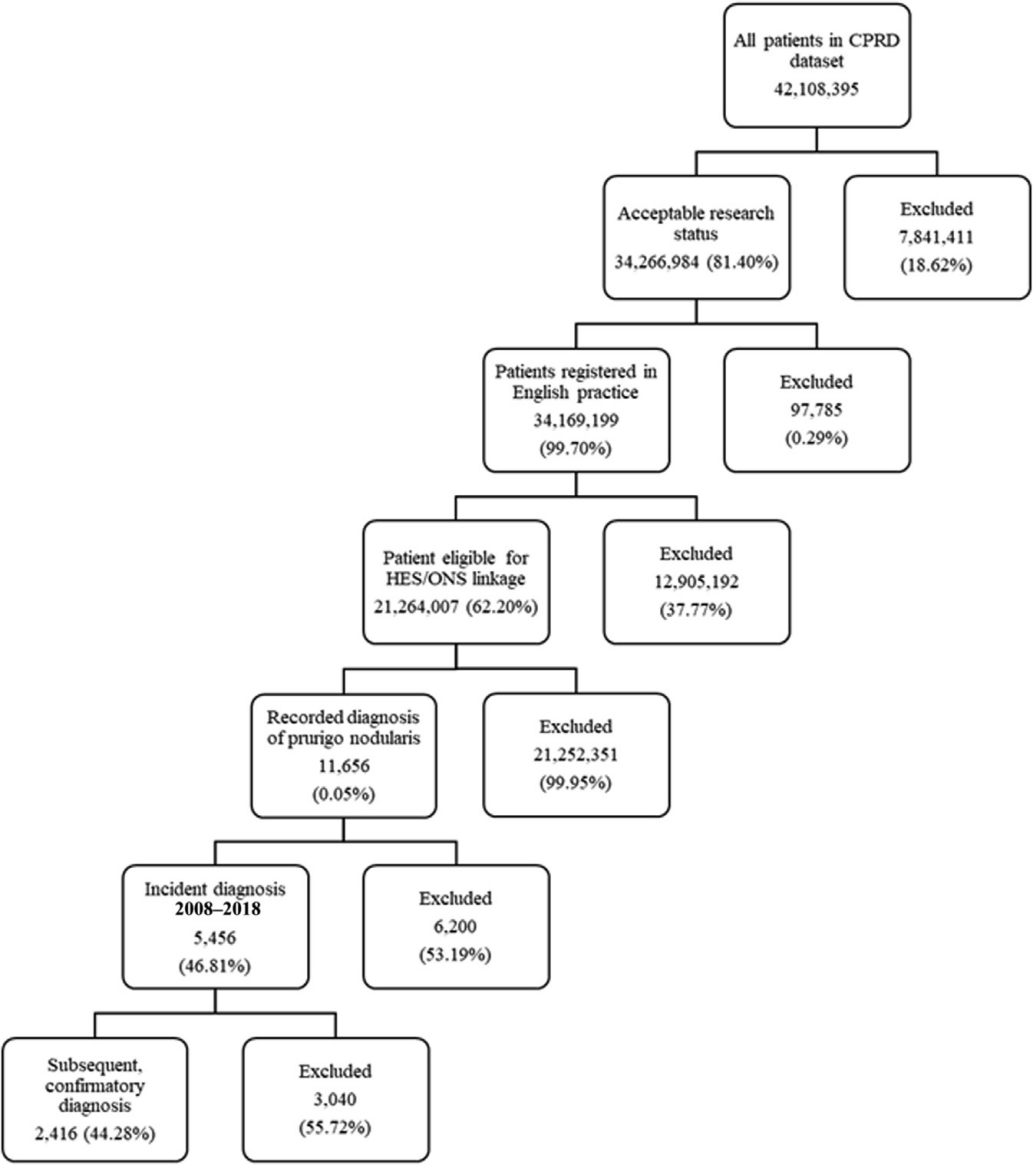
**Derivation of the 2008–2018 incident population with prurigo nodularis from the CPRD Aurum database.** Flowchart showing the derivation of study cohort after application of inclusion and exclusion criteria. CPRD, Clinical Practice Research Datalink; HES, Hospital Episode Statistics; ONS, Office of National Statistics.

There were 42,108,395 patient records in the June 2020 build of Clinical Practice Research Datalink (CPRD). After application of relevant study criteria and Hospital Episode Statistics (HES) linkage eligibility, this decreased to 21,264,007 patients, of whom 11,656 had a relevant diagnosis for PN ever recorded in the dataset. A total of 5,456 (46.81%) were classified as incident cases with a first diagnosis between 2008 and 2018. Of these, 2,416 (48.3%) had a second confirmatory PN diagnosis and formed the main study cohort (Figure 1). The mean duration between the first and second diagnosis was 279 days (median = 78, interquartile range = 25–308). A total of 2,409 (99.7%) patients in the main study cohort and 5,441 (99.7%) in the sensitivity cohort could be matched to controls.

The mean age was 61.0 (SD = 18.4) years, and the majority of patients were female (59.4%). This was similar to the sensitivity cohort who had a mean age of 60.0 (SD = 19.0) years, and 57.2% were female. Owing to the nature of the matching criteria, there was no significant difference between cases and controls in age or gender (Table 1).

**Table 1:** Baseline Demographics of Patients with Prurigo Nodularis and Controls in the Clinical Practice Research Datalink, 2008–2018

Demographics	Main Cohort				Sensitivity Cohort			
	Cases		Controls		Cases		Controls	
Number	2,409		2,409		5,441		5,441	
Follow-up years, mean (SD)	4.11	2.87	4.20	3.03	3.94	2.87	4.08	2.96
Age, y, mean (SD)	61.0	(18.4)	60.9	(18.4)	60.0	(18.9)	59.9	(18.9)
Gender								
Males, n (%)	978	(40.6%)	978	(40.6%)	2,329	(42.8%)	2,329	(42.8%)
Females, n (%)	1,431	(59.4%)	1,431	(59.4%)	3,112	(57.2%)	3,112	(57.2%)
Region, n (%)								
East Midlands	39	(1.6%)	39	(1.6%)	106	(1.9%)	106	(1.9%)
East of England	122	(5.1%)	122	(5.1%)	299	(5.5%)	299	(5.5%)
London	331	(13.7%)	331	(13.7%)	770	(14.2%)	770	(14.2%)
North East	98	(4.1%)	98	(4.1%)	196	(3.6%)	196	(3.6%)
North West	544	(22.6%)	544	(22.6%)	1,158	(21.3%)	1,158	(21.3%)
South Central	211	(8.8%)	211	(8.8%)	542	(10.0%)	542	(10.0%)
South East Coast	141	(5.9%)	141	(5.9%)	377	(6.9%)	377	(6.9%)
South West	310	(12.9%)	310	(12.9%)	679	(12.5%)	679	(12.5%)
West Midlands	503	(20.9%)	503	(20.9%)	1,095	(20.1%)	1,095	(20.1%)
Yorkshire and the Humber	110	(4.6%)	110	(4.6%)	217	(4.0%)	217	(4.0%)

### Prevalent comorbidity

Compared with those in the controls, all selected comorbidities at baseline were significantly increased in patients with a diagnosis of PN with the exception of cancer and HIV, which had fewer than five observations in the controls and was thus suppressed in accordance with CPRD guidelines (Table 2). Atopic dermatitis (AD) was noticeably prevalent with a diagnosis recorded for over half of patients with PN, a 2.5-fold relative risk (RR) increase compared with controls (RR = 2.571; 95% confidence interval [CI] = 2.356–2.806). Other increases were observed for depression (RR = 1.705; 95% CI = 1.566–1.856), anxiety (RR = 1.540; 95% CI = 1.407–1.686), coronary heart disease (CHD) (RR = 1.575; 95% CI = 1.388–1.787), CKD (RR = 1.529; 95% CI = 1.329–1.759), and type 2 diabetes mellitus (T2DM) (RR = 1.836; 95% CI = 1.597–2.111). Similar increases were observed in the sensitivity analysis, which also reported a significant increase in HIV infection (RR = 2.857; 95% CI = 1.209–6.751).

**Table 2:** Comparison of Baseline Comorbidity for Prurigo Nodularis Cohort and Matched Controls in the Clinical Practice Research Datalink, 2008–2018

Outcome	Cases		Controls		Rate Ratio (95% CI)	*P*-Value
Main analysis						
Patients	2,409		2,409			
Atopic dermatitis, n (%)	1,265	(52.5%)	492	(20.4%)	2.571 (2.356–2.806)	<0.0001
Depression, n (%)	999	(41.5%)	586	(24.3%)	1.705 (1.566–1.856)	<0.0001
Anxiety, n (%)	858	(35.6%)	557	(23.1%)	1.540 (1.407–1.686)	<0.0001
HIV, n (%)	6	(0.2%)	<5	—	—	—
Coronary heart disease, n (%)	515	(21.4%)	327	(13.6%)	1.575 (1.388–1.787)	<0.0001
Chronic kidney disease, n (%)	425	(17.6%)	278	(11.5%)	1.529 (1.329–1.759)	<0.0001
Cancer, n (%)	412	(17.1%)	374	(15.5%)	1.102 (0.9692–1.252)	0.1387
Hypertension, n (%)	1,209	(50.2%)	1,020	(42.3%)	1.185 (1.115–1.260)	<0.0001
Type 2 diabetes, n (%)	481	(20.0%)	262	(10.9%)	1.836 (1.597–2.111)	<0.0001
Sensitivity analysis						
Patients	5,441		5,441			
Atopic dermatitis, n (%)	2,576	(47.3%)	1,046	(19.2%)	2.449 (2.305–2.603)	<0.0001
Depression, n (%)	2,090	(38.4%)	1,269	(23.3%)	1.642 (1.549–1.741)	<0.0001
Anxiety, n (%)	1,824	(33.5%)	1,220	(22.4%)	1.502 (1.412–1.597)	<0.0001
HIV, n (%)	20	(0.4%)	7	(0.1%)	2.857 (1.209–6.751)	0.01244
Coronary heart disease, n (%)	1,039	(19.1%)	708	(13.0%)	1.465 (1.342–1.599)	<0.0001
Chronic kidney disease, n (%)	905	(16.6%)	582	(10.7%)	1.549 (1.406–1.707)	<0.0001
Type 2 diabetes, n (%)	981	(18.0%)	575	(10.6%)	1.663 (1.512–1.829)	<0.0001
Cancer, n (%)	910	(16.7%)	811	(14.9%)	1.122 (1.029–1.224)	0.009
Hypertension, n (%)	2,573	(47.3%)	2,189	(40.2%)	1.175 (1.126–1.227)	<0.0001

Abbreviation: CI, confidence interval.

### Incident comorbidity

After the index date, the risks of AD (hazard ratio [HR] = 3.33; 95% CI = 2.55–4.35), depression (HR = 1.61; 95% CI = 1.30–1.99), hypertension (HR = 1.46; 95% CI = [1.19–1.78) and CHD (HR = 1.37; 95% CI = 1.09–1.74) were significantly increased in patients with PN compared with those in controls at α < 0.05 (Table 3). In the sensitivity analysis, with greater statistical power, all a priori selected comorbidities with the exception of cancer were increased.

**Table 3:** Comparison of Incident Morbidity after Index Date for Incident Prurigo Nodularis Cohort and Matched Controls in the Clinical Practice Research Datalink, 2008–2018

Outcome	Prurigo Nodularis			Controls				
	At Risk	Events	Rate ppy	At Risk	Events	Rate ppy	Hazard Ratio (95% CI)^1^	*P*-Value
Main analysis								
Atopic dermatitis	1,144	163	40.6	1,917	87	11.0	3.33 (2.55–4.35)	≤0.0001
Depression	1,410	193	33.1	1,823	152	20.1	1.61 (1.30–1.99)	≤0.0001
Anxiety	1,551	49	7.4	1,852	35	4.5	1.48 (0.95–2.30)	0.0821
Coronary heart disease	1,894	173	21.7	2,082	126	14.5	1.37 (1.09–1.74)	0.0082
Chronic kidney disease	1,984	182	21.9	2,131	137	15.6	1.21 (0.96–1.52)	0.1002
Type 2 diabetes	1,928	129	16.1	2,147	107	12.1	1.25 (0.97–1.63)	0.0888
Cancer	1,997	198	24.4	2,035	203	23.7	1.04 (0.85–1.28)	0.6732
Hypertension	1,200	230	48.8	1,389	185	33.2	1.46 (1.19–1.78)	0.0002
Sensitivity analysis								
Atopic dermatitis	2,865	350	33.8	4,395	214	12.3	2.64 (2.22–3.14)	≤0.0001
Depression	3,351	372	28.0	4,172	295	17.6	1.54 (1.32–1.80)	≤0.0001
Anxiety	3,617	110	7.5	4,221	70	4.1	1.70 (1.25–2.30)	0.0007
Coronary heart disease	4,402	359	20.1	4,733	264	13.7	1.37 (1.16–1.61)	0.0002
Chronic kidney disease	4,536	352	19.3	4,859	277	14.1	1.31 (1.12–1.54)	0.0008
Type 2 diabetes	4,460	279	15.6	4,866	214	10.8	1.34 (1.12–1.61)	0.0014
Cancer	4,531	437	24.9	4,630	438	23.4	1.06 (0.92–1.21)	0.4327
Hypertension	2,868	482	44.5	3,252	420	32.9	1.35 (1.18–1.54)	≤0.0001

Abbreviations: CI, confidence interval; ppy, per patent year.

1 Adjusted for age, gender, and other baseline comorbidities (atopic dermatitis, depression, anxiety, HIV infection, chronic kidney disease, coronary heart disease, cancer, hypertension, and type 2 diabetes mellitus).

### Healthcare resource utilization

Patients with PN had increased primary care contacts relative to controls with 14.8 versus 8.9 contacts per year. Similar excesses were observed in secondary care, with 1.3 versus 0.5 inpatient contacts, 7.6 versus 3.0 outpatient contacts, and 0.6 versus 0.4 accident and emergency contacts (Table 4). After adjusting for baseline characteristics, including baseline morbidity, there were significantly increased incidence rate ratios (IRRs) for all categories of healthcare contacts: IRR = 1.48 (95% CI = 1.47–1.49) for primary care, IRR = 1.80 (95% CI = 1.75–1.85) for inpatients, IRR = 2.15 (95% CI = 2.13–2.18) for outpatients, and IRR = 1.32 (95% CI = 1.27–1.36) for accident and emergency. Similar observations followed in the sensitivity analysis.

**Table 4:** Number, Rate, and Adjusted Rate Ratio of Primary and Secondary Care Contacts for Patients with Prurigo Nodularis Compared with Those for Controls in the Clinical Practice Research Datalink, 2008–2018

Healthcare Sector	Prurigo Nodularis		Controls		Incidence Rate Ratio^1^		
	Contacts	Rate ppy	Contacts	Rate ppy	Rate Ratio	95% CI	*P*-Value
Main analysis							
Patients	2,409		2,409				
All contacts							
Primary care contacts, n (rate ppy)	150,140	14.77	90,981	8.94	1.48	1.47–1.49	<0.0001
Inpatient contacts, n (rate ppy)	13,235	1.30	4,677	0.46	1.80	1.75–1.85	<0.0001
Outpatient contacts, n (rate ppy)	77,683	7.64	30,956	3.04	2.15	2.13–2.18	<0.0001
Accident and emergency contacts, n (rate ppy)	6,431	0.63	3,539	0.35	1.32	1.27–1.36	<0.0001
Prurigo related contacts							
Primary care contacts, n (rate ppy)	6,752	0.66					
Inpatient contacts with primary diagnosis, n (rate ppy)	324	0.03					
Inpatient contacts with any diagnosis, n (rate ppy)	653	0.06					
Dermatology outpatient contacts, n (rate ppy)	26,025	2.56					
Sensitivity analysis							
Patients							
All contacts	5,441		5,441				
Primary care contacts, n (rate ppy)	299,115	13.61	192,598	8.62	1.43	1.42–1.44	<0.0001
Inpatient contacts, n (rate ppy)	25,560	1.16	10,184	0.46	1.72	1.68–1.75	<0.0001
Outpatient contacts, n (rate ppy)	143,004	6.51	66,084	2.96	1.90	1.88–1.92	<0.0001
Accident and emergency contacts, n (rate ppy)	12,679	0.58	7,600	0.34	1.28	1.25–1.31)	<0.0001
Prurigo related contacts							
Primary care contacts, n (rate ppy)	8,551	0.39					
Inpatient contacts with primary diagnosis, n (rate ppy)	429	0.02					
Inpatient contacts with any diagnosis, n (rate ppy)	761	0.03					
Dermatology outpatient contacts, n (rate ppy)	37,298	1.70					

Abbreviations: CHD, coronary health disease; CI, confidence interval; CKD, chronic kidney disease; ppy, per patent year; T2DM, type 2 diabetes mellitus.

1 Adjusted for age at index, gender, baseline CHD, baseline T2DM, baseline CKD, and baseline depression.

Of primary care contacts, 6,752 (4.5%) were coded with a diagnosis of PN. A total of 324 (2.4%) inpatient admissions were coded with PN as the primary diagnosis, and 653 (4.9%) had PN recorded in any position. A total of 26,025 (33.5%) outpatient appointments were in the dermatology specialty.

### Healthcare resource costs

The increase in contacts was also reflected in healthcare costs, with higher mean costs per annum for all healthcare contacts: £371versus £218 for primary care, £1,326 versus £731 for inpatient contacts, £737 versus £331 for outpatient contacts, and £97 versus £53 for accident and emergency contacts (Table 5). Respective cost ratios were 1.78 (95% CI = 1.67–1.90), 1.52 (95% CI = 1.20–1.94), 2.34 (95% CI = 2.13–2.58), and 1.55 (95% CI = 1.33–1.80). Total costs were £2,531 versus £1,333—a cost ratio of 1.62 (95% CI = 1.36–1.94).

**Table 5:** Cost, Cost ppy, and Adjusted Cost Ratio of Primary and Secondary Care Contacts for Patients with Prurigo Nodularis Compared with Those for Controls in the Clinical Practice Research Datalink, 2008–2018

Healthcare Sector	Prurigo Nodularis		Controls		Cost Ratio^1^		
	Total Costs	Cost ppy	Total Costs	Cost ppy	Cost Ratio	95% CI	*P*-Value
Main analysis							
Patients	2,409		2,409				
All contacts							
Primary care contacts, n (rate ppy)	£3,773,115	£371	£2,220,494	£218	1.78	1.67–1.90	<0.0001
Inpatient contacts, n (rate ppy)	£13,486,597	£1,326	£7,439,281	£731	1.52	1.20–1.94	0.0006
Outpatient contacts, n (rate ppy)	£7,498,023	£737	£3,368,008	£331	2.34	2.13–2.58	<0.0001
Accident and emergency contacts, n (rate ppy)	£981,904	£97	£535,574	£53	1.55	1.33–1.80	<0.0001
Total costs	£25,739,639	£2,531	£13,563,357	£1,333	1.62	1.36–1.94	<0.0001
Prurigo related contacts							
Primary care contacts, n (rate ppy)	£214,104	£21					
Inpatient contacts with primary diagnosis, n (rate ppy)	£135,975	£13					
Inpatient contacts with any diagnosis, n (rate ppy)	£1,065,557	£105					
Dermatology outpatient contacts, n (rate ppy)	£1,879,946	£185					
Sensitivity analysis							
Patients	5,441		5,441				
All contacts							
Primary care contacts, n (rate ppy)	£7,541,558	£343	£4,740,449	£212	1.72	1.65–1.80	<0.0001
Inpatient contacts, n (rate ppy)	£26,990,685	£1,228	£15,675,296	£702	1.6	1.36–1.90	<0.0001
Outpatient contacts, n (rate ppy)	£14,166,884	£645	£7,060,972	£316	2.1	1.97–2.24	<0.0001
Accident and emergency contacts, n (rate ppy)	£1,950,609	£89	£1,153,521	£52	1.55	1.40–1.70	<0.0001
Total costs	£50,649,736	£2,305	£28,630,238	£1,281	1.61	1.45–1.80	<0.0001
Prurigo related contacts							
Primary care contacts, n (rate ppy)	£274,022	£12					
Inpatient contacts with primary diagnosis, n (rate ppy)	£198,264	£9					
Inpatient contacts with any diagnosis, n (rate ppy)	£1,303,202	£59					
Dermatology outpatient contacts, n (rate ppy)	£2,721,617	£124					

Abbreviations: CHD, coronary health disease; CI, confidence interval; CKD, chronic kidney disease; ppy, per patent year; T2DM, type 2 diabetes mellitus.

1 Adjusted for age at index, gender, baseline CHD, baseline T2DM, baseline CKD, and baseline depression.

### Mortality

**Figure 2:**
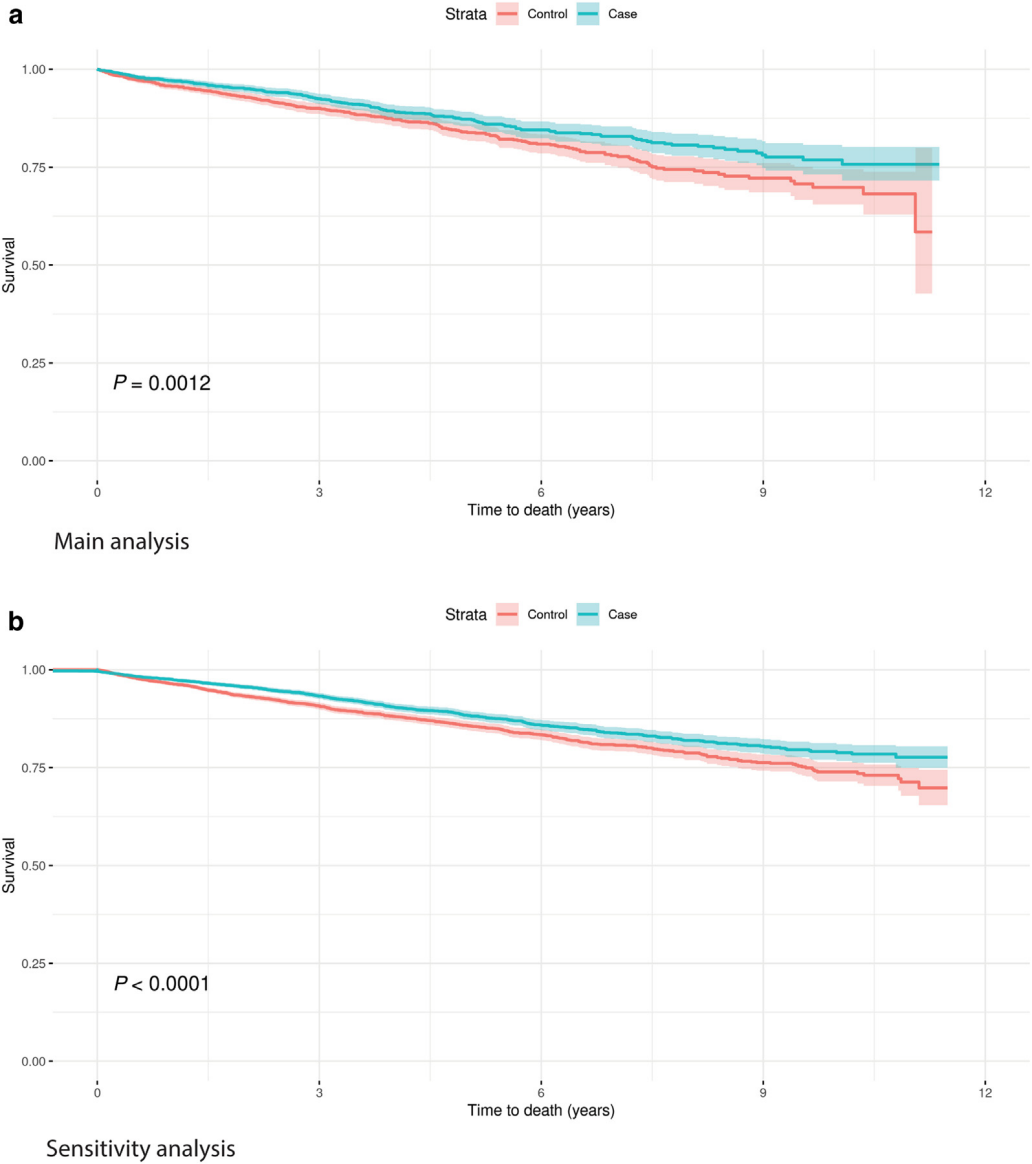
**Kaplan–Meier curve for mortality for patients with prurigo nodularis and controls in the Clinical Practice Research Datalink, 2008–2018.** Kaplan–Meier curve shows time from the index date until death or censorship for patients and matched controls for the main and sensitivity cohorts.

During the follow-up period, there were 284 deaths in those with PN compared with 223 in the controls—crude mortality rates of 36.2 versus 27.1 per 1,000 patient-years, respectively (Table 6 and Figure 2). After adjusting for baseline covariates in a Cox proportional hazards model, the HR was 1.37 (95% CI = 1.14–1.66). In the sensitivity analysis, the HR was 1.28 (95% CI = 1.14–1.44). The underlying cause of death is shown in Table 7, ranked by the most common underlying causes of death in the PN main cohort. The most common underlying cause was chronic ischaemic heart disease recorded for 9.9% of deaths. This was also the most common underlying cause of death in the controls recorded for 7.6% of deaths. No deaths were recorded with PN as the underlying cause of death, and fewer than five had PN recorded as any cause.

**Table 6:** Deaths, Crude Rate, and Adjusted Hazard Ratio for Mortality for Patients with Prurigo Nodularis and Controls in the Clinical Practice Research Datalink, 2008–2018

Cohort	Patients	Deaths	Rate ppy	Hazard Ratio (95% CI)^1^	*P*-Value
Main analysis					
Prurigo nodularis	2,200	284	36.2	1.37 (1.14–1.66)	0.0009
Controls	2,220	223	7.1		
Sensitivity analysis					
Prurigo nodularis	5,441	673	31.4	1.28 (1.46–1.44)	≤0.0001
Controls	5,441	539	24.3		

Abbreviations: CI, confidence interval; ppy, per patent year.

1 Adjusted for age at index, gender, atopic dermatitis, depression, anxiety, HIV infection, chronic kidney disease, coronary heart disease, cancer, hypertension, and type 2 diabetes mellitus.

**Table 7:** Most Common Underlying Causes of Deaths for Patients with Prurigo Nodularis with Comparative Data from Matched Controls

Cause of Death	Main Analysis				Sensitivity Analysis			
	Prurigo Nodularis		Controls		Prurigo Nodularis		Controls	
	Deaths %		Deaths %		Deaths %		Deaths %	
Chronic ischaemic heart disease	28	9.9%	17	7.6%	49	7.3%	46	8.5%
Other chronic obstructive pulmonary disease	22	7.7%	7	3.1%	46	6.8%	25	4.6%
Acute myocardial infarction	15	5.3%	13	5.8%	42	6.2%	23	4.3%
Pneumonia, organism unspecified	11	3.9%	13	5.8%	34	5.1%	28	5.2%
Unspecified dementia	11	3.9%	9	4.0%	32	4.8%	30	5.6%
Malignant neoplasm of bronchus and lung	10	3.5%	7	3.1%	20	3.0%	26	4.8%
Alzheimer's disease	9	3.2%	15	6.7%	21	3.1%	24	4.5%
Atrial fibrillation and flutter	8	2.8%	5	2.2%	15	2.2%	8	1.5%
Heart failure	7	2.5%	<5	—	14	2.1%	7	1.3%
Vascular dementia	7	2.5%	<5	—	13	1.9%	11	2.0%
Malignant neoplasm of colon	7	2.5%	<5	—	12	1.8%	9	1.7%
Stroke, not specified as hemorrhage or infarction	6	2.1%	8	3.6%	12	1.8%	13	2.4%
Other	149	49.6%	—	—	363	53.9%	289	53.6%
Total	284	100.0%	223	100.0%	673	100.0%	539	100.0%

## Discussion

In this retrospective database study of patients with incident PN in England, we report relatively high levels of incident disease comorbidity, resource use, and costs associated with the condition. In the main analysis, for all a priori selected morbidities with the exception of HIV, there were significant increases compared with those of controls at baseline. After the index date, for those patients with no prior history of each specific condition, subsequent incidence of AD, depression, and CHD was significantly increased (at α < 0.05) in patients with PN. These findings provide further support for recent studies highlighting PN as an inflammatory skin disease with associated systemic inflammation (Belzberg et al., 2021; Sutaria et al., 2022b).

The increase in CKD at baseline (1.529; 95% CI = 1.329–1.759) is of interest and comparable with that reported from a US claims database study that reported the OR of 1.85 for patients with CKD overall, increasing to 4.88 for those receiving dialysis, a finding also observed in case reports (Bae et al., 2014; Neild et al., 2011) and also in several additional population-based studies (Huang et al., 2020c; Wongvibulsin et al., 2021). This may be due to the increased levels of uremia associated with severity of CKD and thus hemodialysis acting as a marker of severity. The increase in prevalence of CHD (1.575; 95% CI = 1.388–1.787) and T2DM (1.836; 95% CI = 1.597–2.111) was also observed in the US, with respective ORs of 1.76 (95% CI = 1.53–2.03) and 1.42 (95% CI = 1.30–1.55) (Huang et al., 2020b).

We also report relatively high levels of lifetime depression (41.8%) and anxiety (35.7%). This is higher than reported in other contexts (Jørgensen et al., 2017; Ständer et al., 2020) and may partly reflect our case definition, which included both diagnoses and symptoms such as low mood. However, the relative differences of 1.706 (95% CI = 1.575–1.848) for depression and 1.545 (95% CI = 1.418–1.683) for anxiety between cases and controls is similar to findings from the US, which reported ORs of 2.24 (95% CI = −2.05 to 2.46) for mood disorders and 1.93 (95% CI = 1.78–2.09) for anxiety. The same study also reported increased rates of psychiatric hospitalization for patients with PN (Singam et al., 2020).

The occurrence of PN as an early manifestation of HIV has been previously reported (Matthews and Cockerell, 1998). Owing to the small number of prevalent cases within the controls, this analysis was suppressed in the main analysis, but in the sensitivity analyses, we observed a threefold increase in the prevalence of HIV infection. These results support the findings of an association between PN and HIV in previous studies, which occurs more frequently in Black than in Caucasian patients (Bender et al., 2020).

Previous studies (Larson et al., 2019) have reported an increase in cancer in patients with PN. In this study, although there was an increased prevalence of cancer at baseline, this was only significant in the sensitivity analysis with increased statistical power. There was no significant increase in subsequent cancer rates.

There are limited data regarding the resource utilization and associated cost of treating PN. One study in the US estimated that 3.7 of every 100,000 hospital discharges were for patients with PN and that these patients had significantly increased length of stay and cost compared with patients without PN (Huang et al., 2020a). However, this study was based on those admissions with PN actually coded rather than from a linked approach, so the proportion of admissions is likely to be an underestimate, particularly for overall resource utilization. A large excess cost ($3,206 vs. $643) for emergency room contacts for patients with PN relative to those of controls has been reported in the US (Whang et al., 2021). This was largely driven by the proportion of contacts resulting in full inpatient admission, whereas in our study, we considered these as a separate entity. The increase in costs for accident and emergency contacts was therefore more modest with a cost ratio of 1.55 (95% CI = 1.33–1.80), but although this partly reflected the frequency of contacts, the lower rate ratio for these contacts (1.32; 95% CI = 1.27–1.36) would indicate that once admitted to accident and emergency, patients with PN were more costly to treat.

Overall mean total healthcare costs for patients with PN have been reported as $8,334, with a median of $2,228 accrued over a period of 15 months (Huang et al., 2020a). Adjusting to pound sterling (£1:$1.35) and annualizing, this would amount to £4,938, whereas we report an equivalent figure of £2,531. Within the US study, the majority of excess cost was incurred in the outpatient setting ($5,580 of $8,334), whereas in our study, the majority of costs were from inpatient admissions. However, although only 5% of admissions for our cohort were related to PN, over one third of outpatient appointments were in the dermatology specialty. Although comparisons between different national health contexts are difficult, the cost ratio relative to those of controls in the US study was 1.52, whereas we reported 1.62 in our study.

In this study, we also report a crude mortality rate of 36.2 per 1,000 years. Although not directly comparable owing to differences in the metric, this suggests a mortality profile similar to that observed in Germany (5.4% mortality proportion) (Whang et al., 2021). Adjusting for demographic and morbidity covariates, we estimated 37% higher mortality in those with PN than in controls. These findings of greater mortality in patients with PN confirm a recent multicenter study reporting similar findings (Sutaria et al., 2022a).

As with all retrospective database analyses, there are limitations that should be considered when interpreting results from this study. Although CPRD encompasses both primary and secondary healthcare sources and secondary care sources, there are certain data gaps. Routine data used in this study were primarily collected for the administration of the contributing primary care practices, and data in HES were predominantly collected for reimbursement purposes. This placed certain limitations on this study design. Diagnosis is not routinely recorded in HES outpatient data, so we proxied the burden associated with PN by those appointments within the dermatology specialty, but as is apparent, patients with PN report other dermatological conditions, including AD, both before and after PN diagnosis. Diagnoses may also not be recorded in the electronic records of all primary care contacts, and thus, it is possible that our estimate of primary contacts specifically for PN may be an underestimate.

Presence of morbidities (both as covariates and outcomes) was binary on the basis of the presence or absence of clinical code. We accept that there will be false-negative cases due to under-recording of diagnoses, although by matching on primary practice with the aim to minimize any systematic differences in the recording of diagnoses between primary care practices, which would impact relative rate differences.

We did not conduct a formal validation study to confirm diagnoses in our cohort. A systematic review of validation studies in CPRD (Herrett et al., 2010) reported a median proportion of cases identified of 88.6%, which increased to 94.6% in dermatological conditions. However, validation studies tend to focus on positive predictive value rather than sensitivity, and different diseases are easier to diagnose and classify.

To increase the likelihood of true-positive cases, in the main analysis, we required patients to have had two diagnoses. A previous study in the US (Roh et al., 2022) showed higher true-positive ascertainment associated with multiple diagnoses, although this was based within a tertiary center rather than the predominantly primary care setting used in this study. As with all routine database studies, there will thus be some uncertainty over the accuracy of clinical coding.

In studies with multiple outcomes (Feise, 2002), the chance of falsely rejecting the null hypothesis (a type 1 error) is increased. A level of statistical significance set to the most common threshold (α = 0.05) accepts the likelihood that an apparently significant result would occur by chance if the same experiment was repeated 20 times. Therefore, if we were considering 10 outcomes, it would be likely that at least one significant result would be spurious. Some researchers may adjust α to increase the threshold of statistical significance. One such method is a Bonferroni adjustment, which essentially divides the α by the number of outcomes. For this study, a post hoc Bonferroni adjustment for 13 distinct prospective outcomes (incident comorbidity, resource use, and mortality) would require a significance level of α = 0.004. Such a conservative approach may cause type 2 error, that is, wrongly accepting the null hypothesis, which in this case means that there is no association between the exposure (PN) and the relevant outcome. Furthermore, the Bonferroni correction may be inappropriate where there is a degree of correlation between the outcomes, for example, a patient with a morbidity outcome, such as cancer, is likely to have additional healthcare resource utilization and related costs. In short, there is no universally accepted solution to this issue, but the reader should consider the issue of multiplicity when interpreting the results.

To compare resource use and morbidity, we chose to match patients directly on primary care practice to control for differences in the way that a practice may record data items such as diagnoses. There were also practical considerations in selecting a sample from a control pool rather than from the whole of the CPRD Aurum database. We were able to match 99.7% of cases but accept that we will have discarded data from the non-PN population that was not selected as controls, which could have been included if we had utilized a hierarchical regression method. This may have increased statistical power, especially for rarer outcomes such as HIV infection.

We have described the comorbidity and resource use of an English population with PN and confirmed the findings from other contexts that patients with PN have relatively high levels of comorbidity and consume excess resources. It is of note that comorbidity is increased at diagnosis, and there is a trend toward an excess in the period after diagnosis. The increased prevalence of comorbidity, which may underlie or be consequent to PN, may increase the complexity of management of these patients. Although residual confounding may remain, we estimate that the costs of treating patients with PN after adjusting for baseline morbidity are an excess of 62%. Therapies aimed at reducing inflammation and pruritis associated with PN may reduce resource use as well as improve patient QOL. Furthermore, this population has significant excess mortality.

## Materials and Methods

### Data sources

The study was conducted using the CPRD Aurum database (Wolf et al., 2019). CPRD Aurum contains anonymized patient records from primary care practices in England, including data on demographics, diagnoses, primary care prescriptions, and other aspects of patient care. In addition, patient records can be linked to other datasets, including HES secondary care datasets for inpatients, outpatients, and accident and emergency contacts and the Office of National Statistics mortality data (Office for National Statistics, 2018) containing death registration data. At the time this study was conducted, CPRD Aurum contained currently registered patients for approximately 20% of the English population, and approximately 80% of these patients were linked to the HES and Office of National Statistics datasets. The quality of an individual’s data within CPRD Aurum is flagged as being acceptable for research purposes if the patient records have logical consistency and meet certain criteria such as valid age and gender.

### Study population

The study was restricted to patients from the CPRD Aurum dataset who were defined as being of acceptable research quality and eligible for linkage to the HES secondary care and Office of National Statistics mortality datasets. Cases with PN were defined by clinical code from either the CPRD Aurum observation table (Medcodes 198021000006113 and 3532811000006115 mapping to SNOMED Clinical Terms 105551016 and 3302692015) or from the linked HES Admitted Patients Care inpatient dataset (International Classification of Disease, Tenth Revision code L28.1). Diagnoses are not routinely recorded in the HES outpatient dataset because this does not affect reimbursement. For the main analysis, to exclude false-positive patients whose diagnosis may have been recorded provisionally in primary care prior to referral to secondary care for confirmatory diagnosis, we only included patients with at least two diagnoses of PN recorded on separate occasions in their patient record. In an additional analysis, this restriction was removed. This was to test our assumptions surrounding the main case definition in a sensitivity analysis. In both analyses, the index date was set as the date of the first PN diagnosis.

To ensure that we approximated an incident cohort, patients were required to be registered at the primary care practice for a minimum of 90 days prior to the first recorded diagnosis. To compare morbidity, resource use, and mortality with those of a non-PN population, selected cases were matched at a ratio of 1:1 to patients with no recorded PN diagnosis in their patient records. Patients were matched directly by primary care practice and concurrent practice registration to minimize differences in service provision and biases arising from different preferences in the coding of data items within the database. Additional matching criteria were age (±1 year) and gender.

For the incident morbidity and resource use analyses, patients were followed from the index date to the end of data availability of the HES and Office of National Statistics datasets (June 30, 2019). Patients were censored at the date either the patient was no longer registered at the Aurum primary care practice or the last data collection date for each practice, if that date preceded June 30, 2019. For the mortality analysis, patients in the main cohort were followed from the date of the second confirmatory diagnosis of PN until the end of follow-up.

### Outcomes

On the basis of a review of the literature (Aggarwal et al., 2021; Huang et al., 2020b; Ständer et al., 2020), comorbidities (AD, depression, anxiety, HIV infection, CKD, CHD, cancer, hypertension, and T2DM) were selected. All comorbidities were ascertained by medCODES or International Classification of Disease, Tenth Revision code in the Aurum or HES admitted patient care datasets, respectively.

Primary care contacts were selected from the Aurum consultation table and classified according to the combination of staff role (e.g., general practitioner or practice nurse) and consultation type (e.g., surgery-based consultation, home visit, or telephone consultation). Costs were derived from the Units Costs of Health and Social Care (Curtis and Burns, 2019) on the basis of mapping tables derived internally for previous CPRD studies. Inpatient admissions were ascertained from the HES-admitted patient care dataset. Those admissions with a diagnosis of PN (International Classification of Disease, Tenth Revision L28.1), either as a primary or secondary cause, were flagged and presented separately. Healthcare Resource Groups (HRGs) were assigned to each inpatient admission and processed using HRG4 grouper software (NHS Digital, 2020). The allocated HRGs were then linked to the National Tariff 2019 (NHS England, 2020), adjusting for the nature of the admission (elective admissions vs. emergency) and excess length of stay. Outpatient appointments were ascertained from the HES outpatient dataset. Those appointments within the dermatology specialty were flagged and presented separately. Data were also processed by HRG grouper and mapped to the National Tariff 2019. Accident and emergency appointments were ascertained from the HES Accident and Emergency dataset and processed using HRG4 grouper software and cost on the basis of allocated HRGs linked to the National Tariff 2019.

### Analysis

The incident population was described by age and gender. Lifetime prevalence of each comorbidity at the time of diagnosis was reported for cases and controls, and the RR of cases to controls was calculated.

For patients with no prior history of each comorbidity at baseline, incidence rates of newly diagnosed comorbidities were reported as crude rates. HRs were derived from Cox proportional hazards models adjusting for age, gender, and presence of all other a priori defined covariates (AD, depression, anxiety, HIV infection, CKD, CHD, cancer, hypertension, and T2DM) at the index date.

Rate and cost of inpatient, outpatient, and accident and emergency department utilization per 1,000 person-years were calculated for all patients, with the number of contacts as the numerator and total person follow-up time as the denominator. Rates were compared with those of controls using Poisson regression, and costs were compared using the Gamma distribution. All models were adjusted for age, gender, and presence of AD, depression, anxiety, HIV infection, CKD, CHD, cancer, hypertension, and T2DM at baseline.

A Cox proportional hazards model was constructed to compare time with mortality, adjusting for age, gender, and presence of AD, depression, anxiety, HIV infection, CKD, CHD, cancer, hypertension, and T2DM at baseline. The most common causes of death were also presented and classified by the International Classification of Disease, Tenth Revision diagnosis to the third character. For all analyses, statistical significance was considered at α = 0.05.

This study is based in part on data from the CPRD obtained under license from the United Kingdom Medicines and Healthcare Products Regulatory Agency. The data are provided by patients and collected by the National Health Service as part of their care and support. The interpretation and conclusions contained in this study are those of the author/s alone.

The data used in this study are copyrighted and were reused with permission of the Health and Social Care Information Centre. All rights are reserved.

### Data availability statement

The data that support the findings of this study are available from the Clinical Practice Research Datalink. Restrictions apply to the availability of these data, which were used under license for this study.

## ORCID

Christopher Ll Morgan: http://orcid.org/0000-0001-8796-7406

## Conflict of Interest

CLM, MT, and BRH are employees of Pharmatelligence, a company that provides research services to a range of organizations, including the pharmaceutical industry. Pharmatelligence received funding from Galderma SA to conduct this study. ZKJ-L, CP, SG, and JP are employees of Galderma SA who funded this study. SS has received research grants from Almirall, Beiersdorf, German Research Foundation (DFG), European Academy of Dermatology and Venereology, German Federal Ministry of Education and Research, Interdisciplinary Center for Clinical Research Münster, Leo Pharma, Menlo, Novartis, Sanofi, and Trevi. She has also been an investigator for Celldex, Clexio, Dermasence, Galderma SA, GSK, Kiniksa, Menlo, Trevi, Novartis, and Sanofi and provided consultancy for Abbvie, Almirall, Bayer, Beiersdorf, Bellus Health, Benevolent, Bionorica, Cara, Celgene, CelloHealth, Clexio, DS Biopharma, Eli Lilly, Escient, Galderma SA, Grünenthal, Kiniksa, Klinge Pharma, Menlo, Bayer, Sanofi, Sienna, Trevi, P.G. Unna Academy, Perrigo, Pfizer, Vanda, Vifor, and WebMD. SGK is an advisory board member/consultant for Amgen, Arcutis Biotherapeutics, Aslan Pharmaceuticals, Cara Therapeutics, Castle Biosciences, Genzada Pharmaceuticals, Leo Pharma, Abbvie, Celldex Therapeutics, Galderma SA, Incyte, Johnson & Johnson, Novartis Pharmaceuticals, Pfizer, Regeneron Pharmaceuticals, and Sanofi and has served as an investigator for Galderma SA, Pfizer, and Sanofi.

## References

[bib1] Aggarwal P., Choi J., Sutaria N., Roh Y.S., Wongvibulsin S., Williams K.A. (2021). Clinical characteristics and disease burden in prurigo nodularis. Clin Exp Dermatol.

[bib2] Bae E.H., Park B.M., Kang Y.U., Choi J.S., Kim C.S., Ma S.K. (2014). Prurigo nodularis in a peritoneal dialysis patient. Kidney Int.

[bib3] Belzberg M., Alphonse M.P., Brown I., Williams K.A., Khanna R., Ho B. (2021). Prurigo nodularis is characterized by systemic and cutaneous T helper 22 immune polarization. J Invest Dermatol.

[bib4] Bender A.M., Tang O., Khanna R., Ständer S., Kang S., Kwatra S.G. (2020). Racial differences in dermatologic conditions associated with HIV: a cross-sectional study of 4679 patients in an urban tertiary care center. J Am Acad Dermatol.

[bib5] Curtis L., Burns A. (2019). Unit costs of health & social care. https://www.pssru.ac.uk/project-pages/unit-costs/unit-costs-2019/.

[bib6] Feise R.J. (2002). Do multiple outcome measures require p-value adjustment?. BMC Med Res Methodol.

[bib7] Herrett E., Thomas S.L., Schoonen W.M., Smeeth L., Hall A.J. (2010). Validation and validity of diagnoses in the General Practice Research Database: a systematic review. Br J Clin Pharmacol.

[bib8] Huang A.H., Canner J.K., Kang S., Kwatra S.G. (2020). Analysis of real-world treatment patterns in patients with prurigo nodularis. J Am Acad Dermatol.

[bib9] Huang A.H., Canner J.K., Khanna R., Kang S., Kwatra S.G. (2020). Real-world prevalence of prurigo nodularis and burden of associated diseases. J Invest Dermatol.

[bib10] Huang A.H., Williams K.A., Kwatra S.G. (2020). Prurigo nodularis: epidemiology and clinical features. J Am Acad Dermatol.

[bib11] Huang A.H., Canner J.K., Williams K.A., Grossberg A.L., Kwatra M.M., Kwatra S.G. (2020). Healthcare resource utilization and payer cost analysis of patients with prurigo nodularis. Br J Dermatol.

[bib12] Jørgensen K.M., Egeberg A., Gislason G.H., Skov L., Thyssen J.P. (2017). Anxiety, depression and suicide in patients with prurigo nodularis. J Eur Acad Dermatol Venereol.

[bib13] Larson V.A., Tang O., Stander S., Miller L.S., Kang S., Kwatra S.G. (2019). Association between prurigo nodularis and malignancy in middle-aged adults. J Am Acad Dermatol.

[bib14] Matthews S.N., Cockerell C.J. (1998). Prurigo nodularis in HIV-infected individuals. Int J Dermatol.

[bib15] Morgan C.L., Thomas M., Ständer S., Jabbar-Lopez Z.K., Piketty C., Gabriel S. (2022). Epidemiology of prurigo nodularis in England: a retrospective database analysis. Br J Dermatol.

[bib16] Neild G.H., García-Agudo R., Manzano R., Camacho E., Aoufi S. (2011). Prurigo nodularis in a woman with stage-4 chronic kidney disease. NDT Plus.

[bib17] NHS Digital (2020). HRG4+ 2019/20 local payment grouper. https://digital.nhs.uk/services/national-casemix-.

[bib18] NHS England (2020). National Tariff Payment System. https://improvement.nhs.uk/documents/4980/1920_National_Tariff_Payment_System.pdf.

[bib19] Office for National Statistics (2018). Deaths registration data. https://www.ons.gov.uk/peoplepopulationandcommunity/birthsdeathsandmarriages/deaths.

[bib20] Pereira M.P., Basta S., Moore J., Ständer S. (2018). Prurigo nodularis: a physician survey to evaluate current perceptions of its classification, clinical experience and unmet need. J Eur Acad Dermatol Venereol.

[bib21] Roh Y.S., Marani M., Choi U., Sutaria N., Parthasarathy V., Deng J. (2022). Validation of International Classification of Diseases Tenth Revision code for prurigo nodularis. J Am Acad Dermatol.

[bib22] Singam V., Patel K.R., Silverberg J.I. (2020). Association of prurigo nodularis and lichen simplex chronicus with hospitalization for mental health disorders in US adults. Arch Dermatol Res.

[bib23] Ständer S., Ketz M., Kossack N., Akumo D., Pignot M., Gabriel S. (2020). Epidemiology of prurigo nodularis compared with psoriasis in Germany: a claims database analysis. Acta Derm Venereol.

[bib24] Sutaria N., Adawi W., Brown I., Parthasarathy V., Roh Y.S., Choi J. (2022). Racial disparities in mortality among patients with prurigo nodularis: a multi-center cohort study. J Am Acad Dermatol.

[bib25] Sutaria N., Alphonse M.P., Marani M., Parthasarathy V., Deng J., Wongvibulsin S. (2022). Cluster analysis of circulating plasma biomarkers in prurigo nodularis reveals a distinct systemic inflammatory signature in African Americans. J Invest Dermatol.

[bib26] Tsianakas A., Zeidler C., Ständer S. (2016). Prurigo nodularis management. Curr Probl Dermatol.

[bib27] Whang K.A., Gabriel S., Chavda R., Kwatra S.G. (2021). Emergency department use by patients with prurigo nodularis in the United States. J Am Acad Dermatol.

[bib28] Whang K.A., Kang S., Kwatra S.G. (2019). Inpatient burden of prurigo nodularis in the United States. Medicines (Basel).

[bib29] Whang K.A., Le T.K., Khanna R., Williams K.A., Roh Y.S., Sutaria N. (2022). Health-related quality of life and economic burden of prurigo nodularis. J Am Acad Dermatol.

[bib30] Wolf A., Dedman D., Campbell J., Booth H., Lunn D., Chapman J. (2019). Data resource profile: Clinical Practice Research Datalink (CPRD) Aurum. Int J Epidemiol.

[bib31] Wongvibulsin S., Sutaria N., Williams K.A., Huang A.H., Choi J., Roh Y.S. (2021). A nationwide study of prurigo nodularis: disease burden and healthcare utilization in the United States. J Invest Dermatol.

